# Bridge to nowhere: A retrospective single-center study on patients using chronic intravenous inotropic support as bridge therapy who do not receive surgical therapy

**DOI:** 10.3389/fcvm.2022.918146

**Published:** 2022-08-30

**Authors:** Anirudh Rao, Manavotam Singh, Mansi Maini, Kelley M. Anderson, Nancy A. Crowell, Paul R. Henderson, Sherry S. Gholami, Farooq H. Sheikh, Samer S. Najjar, Hunter Groninger

**Affiliations:** ^1^Department of Medicine, Georgetown University School of Medicine, Washington, DC, United States; ^2^Section of Palliative Medicine, Department of Medicine, MedStar Washington Hospital Center, Washington, DC, United States; ^3^MedStar Washington Hospital Center, MedStar Heart and Vascular Institute, Washington, DC, United States; ^4^Georgetown University School of Nursing and Health Studies, Washington, DC, United States

**Keywords:** inotropes, Stage D heart failure, palliative care, LVAD, heart transplant

## Abstract

**Background:**

Many patients with advanced heart failure (HF) are administered chronic intravenous inotropic support (CIIS) as bridge to surgical therapy; some ultimately never receive surgery. We aimed to describe reasons patients “crossover” from CIIS as bridge therapy to palliative therapy, and compare end-of-life outcomes to patients initiated on CIIS as palliative therapy.

**Methods:**

Single-institution, retrospective cohort study of patients on CIIS as bridge or palliative therapy between 2010 and 2016; data obtained through review of health records and multi-disciplinary selection meeting minutes, was analyzed using descriptive and inferential statistics.

**Results:**

Of 246 patients discharged on CIIS as bridge therapy, 37 (16%) (male *n* = 28, 76%; African American *n* = 22, 60%) ultimately never received surgery. 67 matched patients on CIIS as palliative therapy were included for analysis (male *n* = 47, 70%; African American *n* = 47, 70%). The most common reasons for “crossover” from CIIS as bridge therapy to palliative therapy were frailty (*n* = 10, 27%), cardiac arrest (*n* = 5, 13.5%), and progressive non-cardiac illnesses (*n* = 6, 16.2%). A similar percentage of patients in the bridge (*n* = 28, 76%) and palliative (*n* = 48, 72%) groups died outside the hospital (*P*=0.66); however, fewer bridge patients received hospice care compared to the palliative group (35% vs 69%, *P* < 0.001). Comparing patients who died in the hospital, bridge patients (*n* = 9; 100%) were more likely to die in the intensive care unit than palliative patients (*n* = 8; 42%) (*P* < 0.001).

**Conclusion:**

Patients on CIIS as bridge therapy who do not ultimately receive surgical therapy “crossover” to palliative intention due to frailty, or development of or identification of serious illnesses. Nevertheless, these “bridge to nowhere” patients are less likely to receive palliative care or hospice and more likely to die in the intensive care unit than patients on CIIS as palliative therapy.

## Introduction

The incidence of heart failure (HF) has been steadily increasing over the past decade, with an estimated prevalence of greater than 8 million adults living with HF in the United States by 2030 (1). Given the improvements in survival with HF, the prevalence of patients living with advanced HF is increasing (2). Treatment options for advanced HF include guideline directed medical and device therapies, heart transplantation (HT), left ventricular assist device (LVAD), continuous intravenous inotropic support (CIIS), and symptom-focused treatments (2). The 2013 American College of Cardiology Foundation/American Heart Association (ACCF/AHA) HF guidelines describe the indications for CIIS: Class IIb recommendation as palliative therapy (Level of Evidence B) and Class IIa recommendation as bridge therapy in patients eligible for LVAD or HT (Level of Evidence B) (3). We previously described the clinical course of patients on CIIS as bridge or palliative therapy, and identified a subpopulation of patients initiated on CIIS as “bridge to decision” to surgical therapy who ultimately did not receive LVAD or HT (henceforth referred to in this manuscript as “CIIS as bridge therapy”) (4). In this manuscript, we describe the reasons that this subpopulation of patients on CIIS as bridge therapy “crossover” to CIIS as palliative therapy. Additionally, we report the impact of the initial treatment strategy on the end-of-life course of patients on CIIS as bridge therapy compared to patients whose treatment strategy from the outset was CIIS as palliative therapy.

## Methods

With institutional review board approval from the [withholding institutional identifiers], we conducted a retrospective cohort study on adult patients with ACCF/AHA Stage D HF who were discharged on CIIS from [withholding institutional identifiers], an urban, tertiary-care, academic hospital, between 2010 and 2016. Patients were identified at discharge from their index hospitalization for CIIS initiation and stratified by intention of CIIS, as bridge or palliative therapy. At the study institution, the LVAD/HT evaluation process may be mostly completed during the index hospitalization of CIIS initiation or may unfold as an outpatient in the weeks to months following hospital discharge. Patients who were never discharged from the hospital on CIIS and went straight to LVAD/HT were excluded from the study. Regardless of whether a patient had completed their evaluation for LVAD/HT and deemed to be a candidate or had not begun their evaluation, all patients with clear intent to pursue surgical therapy were deemed “bridge” patients. Electronic health record review was conducted to abstract patient demographics, clinical characteristics, and patient outcomes. Furthermore, for patients on CIIS as bridge therapy who did not ultimately receive surgical therapy, we examined electronic records of available LVAD/HT selection committee meeting minutes to determine the primary reason these patients did not receive surgical therapy. At the study institution, the LVAD/HT selection committee consists of cardiothoracic surgeons, advanced heart failure cardiologists, social workers, pharmacists, nutritionists, LVAD/HT nurse coordinators, psychologists, and palliative care specialists. Other medical subspecialists (Nephrology, Infectious Diseases, Pulmonology, Oncology, etc) and clinical bioethicists are invited to contribute, as appropriate.

The reason for crossover from bridge therapy to palliative therapy was unavailable for 8 patients (21.6%) from the earlier years of the cohort (2010–2012) due to use of a previous electronic record system that was decommissioned prior to the study period. Frailty was a clinical diagnosis made by the patient's advanced HF physicians; no structured screening tool was used to diagnose frailty. End-of-life care outcomes, such as location of death and receipt of palliative care or hospice services, were compared to a subset of patients on CIIS initiated as palliative therapy with an approximate 2:1 cohort matched for age, sex, race/ethnicity, and type of cardiomyopathy. Data were reviewed for all patients through the date of death or referral to hospice if date of death was unavailable.

The results of normally distributed continuous variables are displayed as mean (standard deviation [SD]), and variables not normally distributed are presented as medians (inter-quartile ranges [IQR]). Categorical variables are presented as numbers and percentages. Demographic and clinical characteristics, stratified by indication for CIIS at initiation, were compared. Normally distributed variables were analyzed using *t* test, continuous not normally distributed data were analyzed using the Mann-Whitney U test, and categorical data were analyzed using χ^2^ test. SPSS, version 28 was used to perform statistical analyses. Statistical significance was determined at a 2-tailed *P* < 0.05.

## Results

**Figure 1:**
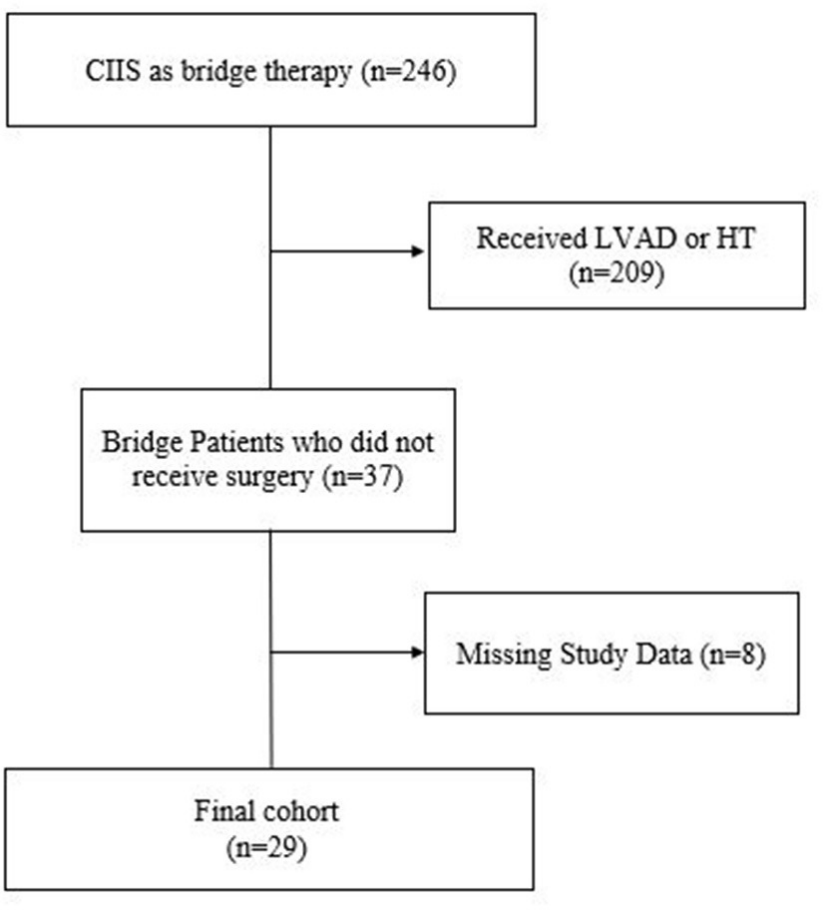
Flow diagram of patients initiated on CIIS as bridge therapy and who ultimately did not receive surgical therapy.

Of the 246 patients discharged on CIIS as bridge therapy, 37 (15%) patients (male *n* = 28, 75.7%; African American *n* = 22, 59.5%) ultimately never received surgery (Figure 1). Sixty-seven patients on CIIS as palliative therapy (male *n* = 47, 70.1%; African American *n* = 47, 70.1%) were matched by age, sex, race, and type of HF. Medical comorbidities were similar between groups except that bridge patients were significantly more likely to have received an implantable cardioverter defibrillator (ICD) prior to initiation of CIIS, compared to patients on CIIS as palliative therapy (97.3 vs. 65.7%; *P* < 0.001) (Table 1).

**Table 1:** Patient demographic characteristics stratified by indication of CIIS at initiation.

	**Bridge therapy (*n* = 37)**	**Palliative therapy (*n* = 67)**	***P***
Age (Median, IQR)	67.3 (52.0–71.3)	68.2 (58.9–78.0)	0.07
Male Sex (n, %)	28 (75.7)	47 (70.1)	0.55
Race (n, %)			0.18
African American/Black	22 (59.5)	47 (70.1)	
White	14 (37.8)	15 (22.4)	
Hispanic/Latino	1 (2.7)	5 (7.5)	
Type of Cardiomyopathy (n, %)			0.42
Ischemic	17 (45.9)	23 (34.3)	
Non-ischemic	18 (48.6)	37 (55.2)	
Mixed ischemic and non-ischemic	2 (5.4)	7 (10.4)	
**Medical Comorbidities (n, %)**			
Diabetes	17 (45.9)	29 (43.3)	0.79
Hypertension	29 (78.4)	46 (68.7)	0.29
Coronary Artery Disease (CAD)	22 (59.5)	36 (53.7)	0.57
Chronic Kidney Disease (CKD)	15 (40.5)	30 (44.8)	0.68
ICD Present	36 (97.3)	44 (65.7)	<0.001

Means compared using independent sample t tests; categorical variables compared using chi squared tests of independence.

ICD, implantable cardioverter defibrillator; IQR, interquartile range.

**Figure 2:**
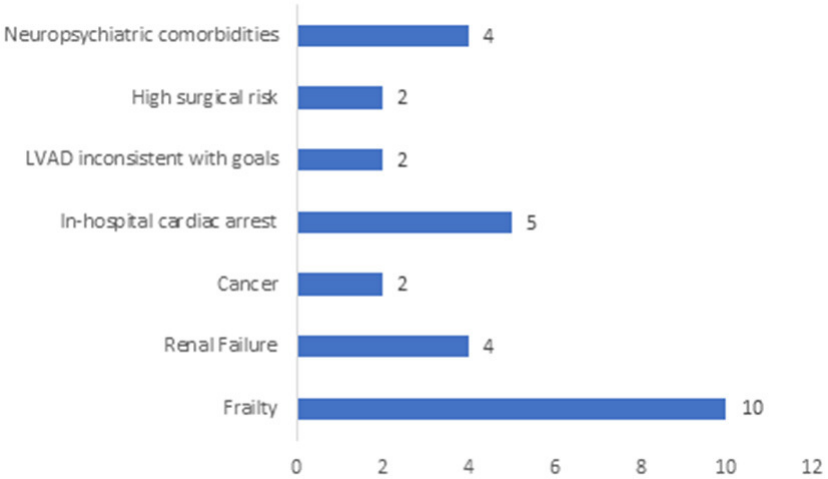
Primary Reason Patient did not Receive LVAD/HT. This figure depicts the primary reason that patients on CIIS as bridge therapy did not ultimately receive surgical therapy for advanced heart failure. Patient data was unavailable for *n* = 8 (22%) of the cohort due to inability to access data from an electronic health record that was decommissioned prior to the study period.

Frailty was the most common reason for crossover from CIIS as bridge therapy to palliative therapy (*n* = 10, 27.0%) followed by medical comorbidities such as progressive renal failure (*n* = 4, 10.8%) or malignancy (*n* = 2, 5.4%). Cardiac arrest accounted for the cause of death for five patients (13.5%). Neuropsychiatric comorbidities became apparent for four patients during the evaluation process, precluding advanced surgical therapies (Figure 2). Data regarding the reasons for reclassification of intention of CIIS from bridge to palliative was unavailable for eight patients.

All 37 patients who received CIIS as bridge therapy died, as did all 67 patients on CIIS as palliative therapy, with a median time from inotrope initiation to death of 4.0 and 3.3 months, respectively (*P* = 0.055). There was no difference in location of death (in-hospital versus out-of-hospital) between patients on CIIS as bridge therapy compared to those on CIIS as palliative therapy (*P* = 0.66). However, among patients who died at the study institution, all patients (*n* = 9, 100%) on CIIS as bridge therapy died in the intensive care unit compared to only 8 patients (42%) in the palliative therapy group (*P* < 0.001). Location of out-of-hospital death could not be confirmed for most patients, although the proportions of patients who died at home with hospice or at inpatient hospice were similar between the two groups. Fewer patients in the bridge therapy group received palliative care consultation (*n* = 13, 35% vs *n* = 52, 77.6%; *P* < 0.001) or referral to hospice (*n* = 13, 35% vs *n* = 46, 68.7%; *P* < 0.001) than the palliative group (Table 2).

**Table 2:** Time from inotrope start to death, location of death, palliative care consultation, and hospice referral stratified by group.

	**Bridge therapy (*n* = 37)**	**Palliative therapy (*n* = 67)**	***P***
Median time in months from inotrope start to death (IQR)	4.0 (2.6–10.8)	3.3 (1.1–7.9)	0.055
In-hospital death, *n (%)*	9 (24.3)	19 (28.4)	0.66
Non-hospital death, *n (%)*	28 (75.7)	48 (71.6)	0.66
In-Hospital Death			<0.001
ICU, *n (%)*	9 (100.0)	8 (42.1)	
Floor, *n (%)*	0 (0.0)	11 (57.9)	
**Non-hospital death**			
Home with hospice, n (%)	7 (25.0)	15 (31.3)	
Inpatient hospice, n (%)	5 (17.9)	14 (29.2)	
Unavailable, n (%)	16 (57.1)	19 (39.6)	
Palliative care consultation	13 (35.1)	52 (77.6)	<.001
Hospice enrollment	13 (35.1)	46 (68.7)	<.001

For non-hospital death, statistical test could not be performed due to the high percentage patients with unavailable data.

IQR, interquartile range; ICU, intensive care unit.

## Discussion

In this retrospective cohort study, we report on the clinical course and causes of death of patients initiated on CIIS as bridge therapy who crossover to CIIS as palliative therapy. The question motivating this study is whether the strategy at time of initiation of therapy affects the treatment course. Our study indicates that the strategy at CIIS initiation influences the end-of-life course of these patients. Patients initiated on CIIS as bridge therapy who did not ultimately receive advanced HF surgical interventions were more likely to die in the intensive care unit, and have less palliative care consultation and hospice referrals, than patients initiated on CIIS *a priori* as palliative therapy alone. For patients who died in-hospital, patients on CIIS as bridge therapy were more likely to die in the intensive care unit compared to patients on CIIS as palliative therapy. In these regards, our study suggests that the initial treatment strategy influences clinical decision making that impacts end-of-life care for patients and families. Similarly, in a recent study of patients with end stage renal disease, patients who were evaluated for kidney transplant received more intensive care at the end-of-life than patients who were not being considered for kidney transplant (5).

Once the risks of surgical therapies for advanced HF are deemed to be prohibitive, patients require a deliberate reclassification of the CIIS treatment strategy with communication to the broader care team in order to transition from a treatment approach of optimizing patients for surgery to one aligned with the patient's prognosis. Additionally, routine involvement of specialist palliative care consultants for *all* patients on CIIS ensures that patient's treatments are contextualized with their prognosis and concordant with their goals of care. Routine inclusion of palliative care specialists also fulfills National Coverage Determinations (NCDs) from Centers for Medicare & Medicaid Services (CMS) for the patients on CIIS as bridge therapy who later receive LVAD as destination therapy. Lastly, since the prognosis on inotropes is similar between the two groups, routine inclusion of palliative care specialists may facilitate earlier identification of patients on CIIS as bridge therapy who do not seem likely to receive surgical therapy to initiate discussions regarding advance care planning. As a result of these factors, we implemented a new model of collaboration in 2015 by embedding palliative care within the Advanced Heart Failure program at the study institution. Since this time, palliative care specialists regularly participate in LVAD/HT selection committee meetings to contribute to the committee's understanding regarding patients' goals of care. Furthermore, consistent participation of palliative care specialists in LVAD/HT selection committee meetings allows for earlier identification of patients who are deemed ineligible for surgical therapies. This gives the palliative care team more time to build rapport, cultivate prognostic awareness, and redirect care away from aggressive treatments toward care that is more appropriate (implementing a Do Not Attempt Resuscitation order, for example).

To our knowledge, this is the first study that describes why patients on CIIS as bridge therapy do not receive surgical therapy for advanced HF, effectively serving as a “bridge to nowhere.” This “bridge to nowhere” concept has been described for patients on extracorporeal membrane oxygenation or other temporary mechanical circulatory supports (6). Guidelines regarding the LVAD and HT evaluation processes provide a thorough framework for the medical and psychosocial evaluations that precede surgery (7–9). This study highlights that a notable percentage of patients initiated on CIIS as bridge therapy (15% in our cohort) undergo a change in their inotrope treatment strategy during the LVAD/HT evaluation process. We hypothesized that most patients who “crossover” from CIIS as bridge therapy to palliative therapy did so because of an acute event, such as a cardiac arrest, septic shock, or cardiogenic shock. However, we demonstrate that, more frequently, the surgical evaluation process uncovers a chronic, progressive, non-cardiac process that makes the risks of surgical therapy prohibitive. Frailty, a multidimensional construct, emerged as the most common reason patients were no longer considered for surgical therapy, likely as part of a constellation of relative contraindications of high-risk features associated with poor outcomes. The AATS/ISHLT guidelines comment on the association between cachexia and poor post-operative outcomes (7), but frailty is a clinical syndrome that is parallel to, and not defined by, cachexia (10, 11), and is associated with poorer outcomes post-LVAD (12, 13). Standardized tools for evaluation of frailty include gait speed testing and hand grip strength testing, (11, 13) though it is unknown how commonly frailty testing is performed as a part of pre-LVAD evaluation (14). The results of our study emphasize the importance of structured assessments of frailty prior to launching the LVAD/HT evaluation process. Once inotrope-dependent patients are deemed to be frail, a structured prehabilitation program with multi-disciplinary input from rehabilitation, nutrition, psychology, palliative care, and cardiovascular experts should be implemented. Subsequent assessments of frailty can identify patients whose frailty phenotype may be reversible. This subset can be evaluated further for suitability for LVAD/HT, whereas patients whose frailty does not improve may be best treated under a palliative paradigm.

Some patients are newly diagnosed with a malignancy during the surgical evaluation process. These patients may merit specialist palliative care consultation to weigh the competing disease processes and the impact on prognosis, and to explore treatment preferences regarding the available treatment options. Neuropsychiatric comorbidities are also likely to be present prior to the decision to initiate CIIS as bridge therapy. Some patients who are in low output HF present with signs of mild cognitive impairment, that may improve with LVAD therapy (15). However, certain patients on CIIS as bridge therapy demonstrate significant neurocognitive impairments that prove prohibitive for consideration of surgical therapy. These patients, who are likely best served under a palliative paradigm, merit additional consideration prior to being designated as bridge therapy patients given the influence that initial indication for CIIS has on end-of-life outcomes.

This study has several limitations. First, the retrospective study design and need for chart review opens the possibility of incomplete characterization of the nuances of each patient's surgical evaluation. Second, since the study examined patients at a single institution, the outcomes described may reflect local practice patterns that have evolved since the era of the study period and may not be generalizable. Some data was unavailable due to the use of an obsolete electronic record system in the earliest years of the study period, and specific location of out-of-hospital death was not known for a plurality of patients in both groups. Lastly, the concept of intention of CIIS therapy as being bridge or palliative may represent a fluid spectrum as opposed to a binary construct.

In conclusion, this study illuminates a subpopulation of patients on CIIS as bridge therapy who do not ultimately receive surgical therapy. The reasons for “crossover” from bridge to palliative intention include new medical conditions, progression of underlying disease states, or acute events. These patients receive more aggressive care at the end-of-life compared to patients on CIIS as palliative therapy. Frailty and neurocognitive impairment likely can be identified through more intentional screening of at-risk groups during the surgical evaluation process. Given the prognostic uncertainty associated with CIIS as bridge therapy, and the disparate end-of-life outcomes of patients on CIIS as bridge vs. palliative therapy, palliative care specialists should be routinely consulted for this population.

## Data availability statement

The raw data supporting the conclusions of this article will be made available by the authors, without undue reservation.

## Author contributions

AR, MS, MM, PH, and SG were responsible for data collection. AR, KA, FS, SN, and HG contributed significantly to the study design, data analysis, and interpretation. AR, KA, and NC performed statistical analyses. AR, MS, MM, KA, NC, PH, SG, FS, SN, and HG contributed to the manuscript. All authors contributed to the article and approved the submitted version.

## Conflict of interest

The authors declare that the research was conducted in the absence of any commercial or financial relationships that could be construed as a potential conflict of interest.

## Publisher's note

All claims expressed in this article are solely those of the authors and do not necessarily represent those of their affiliated organizations, or those of the publisher, the editors and the reviewers. Any product that may be evaluated in this article, or claim that may be made by its manufacturer, is not guaranteed or endorsed by the publisher.
